# Immunodiagnosis of Paracoccidioidomycosis due to *Paracoccidioides brasiliensis* Using a Latex Test: Detection of Specific Antibody Anti-gp43 and Specific Antigen gp43

**DOI:** 10.1371/journal.pntd.0003516

**Published:** 2015-02-13

**Authors:** Priscila Oliveira dos Santos, Anderson Messias Rodrigues, Geisa Ferreira Fernandes, Silvia Helena Marques da Silva, Eva Burger, Zoilo Pires de Camargo

**Affiliations:** 1 Federal University of São Paulo, Department of Microbiology, Immunology and Parasitology, Cell Biology Division, São Paulo, Brazil; 2 Evandro Chagas Institute, Bacteriology and Mycology Division, Ananindeua, Pará, Brazil; 3 Federal University of Alfenas, Institute of Biomedical Sciences, Alfenas, Minas Gerais, Brazil; University of California San Diego School of Medicine, UNITED STATES

## Abstract

**Background:**

Paracoccidioidomycosis (PCM) is a life-threatening systemic disease and is a neglected public health problem in many endemic regions of Latin America. Though several diagnostic methods are available, almost all of them present with some limitations.

**Method/Principle Findings:**

A latex immunoassay using sensitized latex particles (SLPs) with gp43 antigen, the immunodominant antigen of *Paracoccidioides brasiliensis*, or the monoclonal antibody mAb17c (anti-gp43) was evaluated for antibody or antigen detection in sera, cerebrospinal fluid (CSF), and bronchoalveolar lavage (BAL) from patients with PCM due to *P. brasiliensis*. The gp43-SLPs performed optimally to detect specific antibodies with high levels of sensitivity (98.46%, 95% CI 91.7–100.0), specificity (93.94%, 95% CI 87.3–97.7), and positive (91.4%) and negative (98.9%) predictive values. In addition, we propose the use of mAb17c-SLPs to detect circulating gp43, which would be particularly important in patients with immune deficiencies who fail to produce normal levels of immunoglobulins, achieving good levels of sensitivity (96.92%, 95% CI 89.3–99.6), specificity (88.89%, 95% CI 81.0–94.3), and positive (85.1%) and negative (97.8%) predictive values. Very good agreement between latex tests and double immune diffusion was observed for gp43-SLPs (k = 0.924) and mAb17c-SLPs (k = 0.850), which reinforces the usefulness of our tests for the rapid diagnosis of PCM in less than 10 minutes. Minor cross-reactivity occurred with sera from patients with other fungal infections. We successfully detected antigens and antibodies from CSF and BAL samples. In addition, the latex test was useful for monitoring PCM patients receiving therapy.

**Conclusions/Significance:**

The high diagnostic accuracy, low cost, reduced assay time, and simplicity of this new latex test offer the potential to be commercialized and makes it an attractive diagnostic assay for use not only in clinics and medical mycology laboratories, but mainly in remote locations with limited laboratory infrastructure and/or minimally trained community health workers.

## Author Summary

Paracoccidioidomycosis is one of the most prevalent systemic mycoses in Latin America, and still poses a significant threat to the health of human hosts, especially those with an impaired immune system. Early and accurate diagnosis is mandatory for the implementation of effective treatment. Currently, most of the diagnostic tests are not simple to implement in areas where laboratory infrastructure or trained personnel are not available. To overcome this problem, we propose a simple and inexpensive assay for use in latex agglutination tests based on the *P*. *brasiliensis*-specific antigen gp43 or monoclonal antibody (mAb17c) anti-gp43 coupled to latex particles in order to detect both circulating antigens and antibodies in sera, cerebrospinal fluid, and bronchoalveolar lavage. Our results show that PCM can be diagnosed with high sensitivity and specificity in less than 10 min using these tools. In addition, the latex test demonstrated its applicability in the follow-up of PCM patients during antimycotic therapy. The diagnostic accuracy, low cost, and simplicity of this fast test makes it an attractive serological assay that can be implemented in endemic areas with remote access, in laboratories with limited infrastructure, and/or to the community health agents trained to use it in rural areas.

## Introduction

Neglected tropical diseases (NTDs), a group of chronic diseases caused by debilitating parasitic, bacterial, viral, and fungal infections are among the most common causes of illness in the poorest people living in developing countries [1]. Fungal infections represent an important health problem in lower income countries. Paracoccidioidomycosis (PCM) is a systemic endemic mycotic disease affecting mainly male rural workers during the most productive years of their life, which poses a significant public health issue and causes important economic losses in Latin America; this epidemiological scenario tends to concentrate around humid forests in subtropical and tropical areas [2]. PCM has an estimated incidence of one to three cases per 100,000 inhabitants, the majority occurring in Brazil, Colombia, and Venezuela, where the fungus is endemic [3,4].

Multi-locus sequencing studies have clarified species boundaries within etiological agents of *Paracoccidioides* and support the existence of clinically important cryptic groups beyond *Paracoccidioides brasiliensis* [5]. The *P*. *brasiliensis* complex comprises four genetic groups of clinical interest, including species 1 (S1), phylogenetic species 2 (PS2), phylogenetic species 3 (PS3), and phylogenetic species 4 (PS4) [3,5,6]. A sister taxa referred to as a new biological species, *Paracoccidioides lutzii*, is placed at a relatively large distance from the *P*. *brasiliensis* complex by phylogenetic analysis [7,8]. Epidemiological studies support a broad range for the agents embedded in the *P*. *brasiliensis* complex, especially the S1 group, which is predominant in Latin America, whereas the offshoot *P*. *lutzii* appears to be prevalent in the Brazilian territory, which has an epicenter in the central-west region [9–11] and few cases reported outside this area [12], but its real incidence is unknown [13].

Disease acquisition involves inhalation of *Paracoccidioides* propagules from the environment leading to a primary pulmonary infection with no latency period, or more commonly the reactivation of quiescent foci [14]. Patients present with variable clinical manifestations, ranging from an acute/subacute to chronic form. PCM is classically diagnosed by identifying multiple budding yeast cells in biological fluids or histologically by visualizing yeasts in tissue sections [14–16]. However, the detection of the pathogen in biological fluids is often difficult due to the few pathognomonic structures. In addition, cultures are time consuming and not easily obtained, especially from sputum, the material most commonly sent to the laboratory. In the absence of visualizing fungal structures in biological fluids, serological assays such as double immunodiffusion (DID) [17,18], dot-blot [19], ELISA [20,21], Western blot [22], and latex agglutination (LA) [23] have been extremely useful for confirming diagnosis. These tests are used broadly over classical methods due to low cost, reproducibility, and ease of implementation in the laboratory. Of the recommended serological tests, those that demonstrate the presence of circulating antibodies in the sera are the most frequently employed for diagnosis and patient follow-up [24–26]. The *P*. *brasiliensis* immunodominant antigen gp43, a 43,000 Dalton glycoprotein expressed during infection, induces a strong antibody response and has been proposed as an important serological marker because it is recognized by a most PCM sera due to *P*. *brasiliensis* [22,27].

Despite continuous improvements in immunological tools for the diagnosis of PCM, the techniques used for primary diagnosis, at least in field situations, still rely on direct observation of the fungal structures in biological fluids. Tissue forms of *P*. *brasiliensis* are similar to *Histoplasma capsulatum* and may lead to misdiagnosis; for accurate diagnosis the section often has to be examined carefully to determine the pathognomonic stages of the fungus. Therefore, *Paracoccidioides* infections need to be diagnosed rapidly, especially among populations living in neglected areas. In this scenario the LA tests are very popular in clinical laboratories for the diagnosis of viral, bacterial, fungal, and parasitic diseases [28].

A rapid and simple latex test to detect and monitor antigens and antibodies in serum samples is overdue in routine field practice, especially for subjects living in neglected areas. Due to the high incidence of PCM caused by *P*. *brasiliensis* in Latin America (S1, PS2, and PS3), the present study was designed to standardize a LA test using purified gp43 antigen and anti-gp43 monoclonal antibody coupled to latex particles to evaluate the potential capacity for the detection of specific anti-gp43 antibodies or gp43 antigen in sera, cerebrospinal fluid (CFS), and bronchoalveolar lavage (BAL). Moreover, sera from PCM patients receiving antifungal therapy were followed up based on the antibody titer and antigen detection measured by the LA test in order to verify its usefulness for monitoring the patients.

## Materials and Methods

### Ethics statement

This study was approved by the Research Ethics Committee of Federal University of São Paulo (UNIFESP). All patients provided informed written consent and the study was approved by the ethical committee under number CEP 1796/10.

### Biological material

Sixty-five serum samples obtained from patients with active PCM (61 males and 4 females, age range 3 to 69 years) were included in this study. Eight patients presented with the acute form of the disease and 57 patients presented with the chronic form. In addition, 14 CSF samples were obtained from neuroPCM patients and 13 samples of BAL fluid from patients with pulmonary PCM. The diagnosis of PCM was confirmed by direct examination of biological fluids and/or serological immunodiffusion tests. Serum samples were obtained from patients with histoplasmosis (n = 18), aspergillosis (n = 18), candidiasis (n = 13), and non-fungal diseases (n = 12), and sera from healthy individuals (n = 38) were used as controls. In addition, six CSF and six BAL samples from patients with other non-fungal diseases were used as controls. All samples were stored at -20°C until use. The undiluted CSF and BAL samples were inactivated at 56°C for 30 minutes before use.

### Clinical samples for monitoring therapy

PCM patients (n = 10) undergoing therapy were evaluated by LA for serological follow-up of antigen and antibody detection. The diagnosis was supported by the clinical experience of the physician responsible for the patient presenting with signs and symptoms of the disease at diagnosis. The patients were selected based on the number of samples in the interval between a pickup and another and the type of treatment used. PCM was confirmed in most patients via direct examination of secretions, such as sputum, oral mucosa lesion samples, or biopsies. Serological ID tests using the traditional exoantigen from *P*. *brasiliensis* B-339 (AgPbB339 standard antigen) confirmed the diagnosis. Five patients presented with the acute form of the disease and five patients presented with the chronic form. Seven patients were treated with itraconazole (200 mg) twice a day and three patients with sulfamethoxazole (400 mg) + trimethoprim (80 mg) twice a day. Patients were evaluated at the moment of diagnosis (T1) and followed up at 3 (T2) and 18–24 months (T3). Three serum samples from each patient were analyzed, for a total of 30 samples. Patients were aged between 3 and 56 years.

### Fungal strain, gp43 antigen, and mAb anti-gp43

*P*. *brasiliensis* B-339 (ATCC 32069; PS3) was obtained from Dr. A. Restrepo (Corporation Investigaciones Biológicas, Medellín, Colombia) and has been maintained on Sabouraud dextrose agar (Difco Laboratories, Detroit, Mich.) in our laboratory since the 1970s. The fungus was converted to the yeast form on modified Sabouraud dextrose agar containing 0.01% thiamine (Difco Laboratories, Detroit, Mich.) and 0.14% asparagine (Difco Laboratories, Detroit, Mich.) (Sab-T-A) at 35°C. Exoantigen from the B-339 strain was used to purify gp43 as described previously [17], submitted to SDS-PAGE [29], and silver stained [30] to verify the purification. Purified protein concentrations were determined by the Bradford method [31] and stored at -20°C until use. The mAb anti-gp-43 (mAb17c) was kindly provided by Dr. R. Puccia [32,33].

### Double immunodiffusion test

Three millimeters of melted 1% agarose (Sigma A-6877) in PBS was poured onto a glass slide (75 × 25 mm). The pattern for this micro-ID test consisted of a central well surrounded by six wells, each 3 mm in diameter. The central well was located 6 mm (edge-to-edge) from the other wells and filled with the antigen solution. Each slide contained two sets of wells. On each slide, the two central wells were filled with 10 μl of antigen. Surrounded wells were filled with diluted serum (1:2 to 1:1024). The slides were incubated in a moist chamber at room temperature (20–25°C) for 48 h, and then washed for 1 h in 5% sodium citrate and 24 h in saline. The slides were dried, stained for 5 min with 0.15% Coomassie Brilliant blue (Sigma) in ethanol:acetic acid:water (4:2:4; v:v), and destained in the solvent mixture alone, when necessary. Precipitation bands were recorded by visual observation [17]. Sera from patients with PCM (n = 65), histoplasmosis (n = 18), aspergillosis (n = 18), candidiasis (n = 13), non-fungal diseases (n = 12), and healthy individuals (n = 38) were tested individually and the titer of each serum sample determined.

### Sensitization of latex particles with gp43 for specific anti-gp43 antibody detection

**Fig 1:**
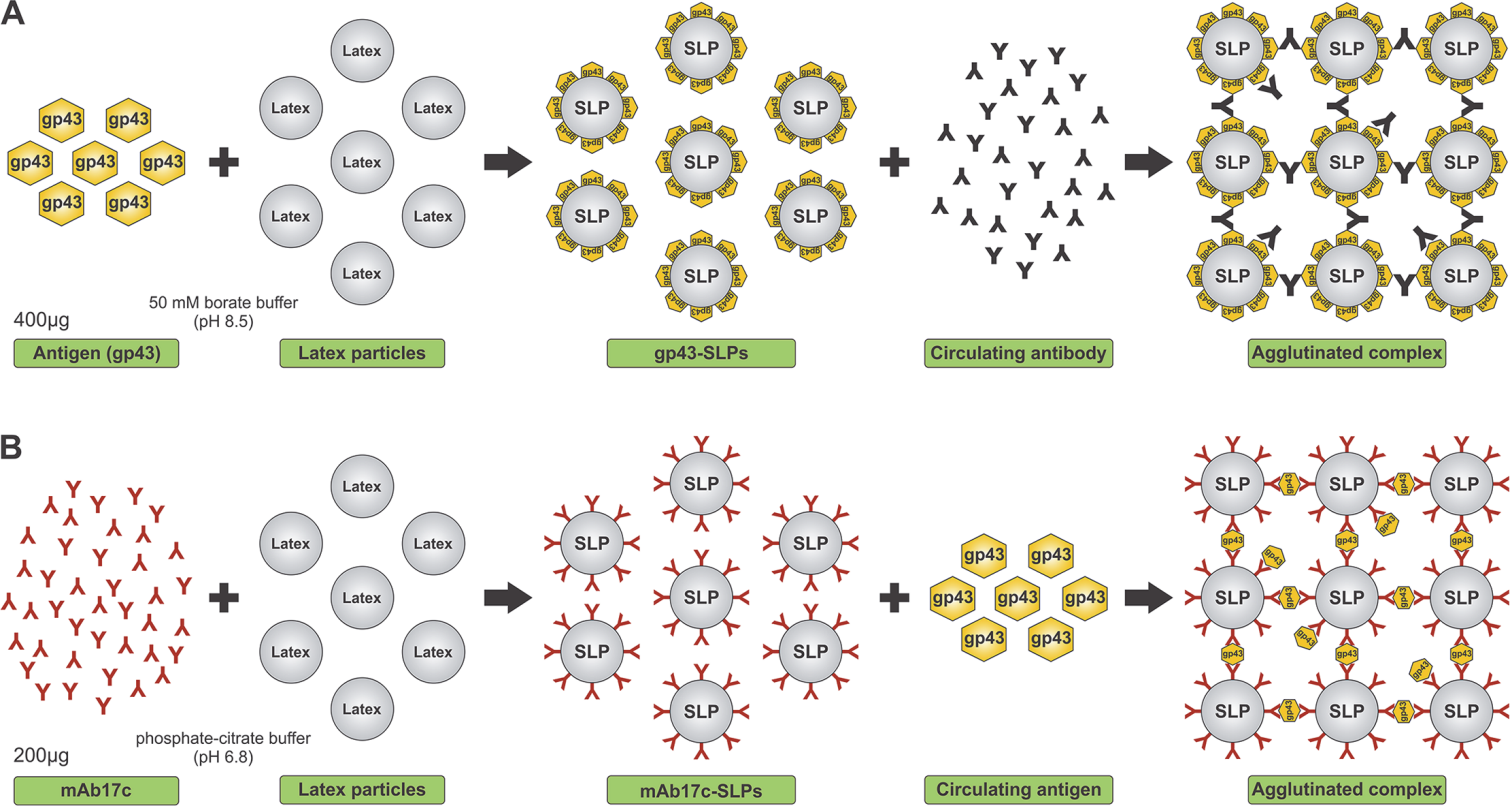
Diagram of latex agglutination. (**A**) Reaction between gp43-sensitized latex particles (SLPs) for detection of specific antibodies and (**B**) between the monoclonal antibody mAb17c-SLPs for detection of circulating gp43 antigen in the sample.

Purified gp43 was coupled to carboxylated latex particles (Carboxyl latex microspheres, Invitrogen) 0.8 μm in diameter according to the manufacturer’s instructions, creating gp43-SLPs (Fig. 1A). Briefly, 250 μl of a 4% (wt/vol) suspension of the particles was washed three times in 750 μl of 50 mM borate buffer (pH 8.5). All washes were done at 10,000 x g for 10 min at room temperature unless otherwise stated. Following the final wash, the particles were suspended in 1 ml of 50 mM borate buffer (pH 8.5), 400 μg of purified gp43 added to the mixture, and then incubated for 24 h at 4°C with gentle end-to-end rotation. The mixture was centrifuged for 10 min at 10,000 x g and the supernatant saved for protein determination [31]. To block non-specific binding sites, the sediment was resuspended in 50 mM phosphate buffered saline (PBS; pH 7.2) containing 1% bovine serum albumin (BSA) fraction V and incubated for 4 h at room temperature (20–25°C) with gentle mixing. After washing three times in 50 mM PBS (pH 7.4), the sensitized latex particles (SLPs) were resuspended in 1 ml of storage buffer (50 mM PBS, pH 7.4, containing 1% BSA, 0.1% NaN_3_, and 5% glycerol) and stored at 4°C until required.

### Sensitization of latex particles with mAb anti-gp43 for detection of gp43

Purified mAb17c was used to coat carboxylated latex particles (Carboxyl latex microspheres, Invitrogen) 0.8 mm in diameter following the manufacturer’s instructions, creating mAb17c-SLPs (Fig. 1B). Briefly, 250 μl of a 4% (wt/vol) suspension of the particles was washed three times with 750 μl of phosphate-citrate buffer (pH 6.8). All washes were done at 10,000 x g for 10 min at room temperature unless otherwise stated. Following the final wash, the particles were suspended in 1 ml of phosphate-citrate buffer (pH 6.8), and 200 μg of mAb17c was added to the mixture and incubated for 24 h at 4°C with gentle end-to-end rotation. The mixture was centrifuged for 10 min at 10,000 x g and the supernatant saved for protein determination [31]. To block non-specific binding sites, the sediment was resuspended in 50 mM PBS (pH 7.2) containing 1% BSA fraction V and incubated for 4 h at room temperature (20–25°C) with gentle mixing. After washing three times in 50 mM PBS (pH 7.4), the SLPs were resuspended in 1 ml of storage buffer (50 mM PBS, pH 7.4, containing 1% BSA, 0.1% NaN_3_, and 5% glycerol) and stored at 4°C until required.

### Latex agglutination assay

The LA test was performed by mixing 25 μl of gp43-SLPs or mAb17c-SLPs with a total of 25 μl of serum, CSF, or BAL sample on a black-coated glass slide. Just for the test using mAb17c-SLPs (for the detection of gp43), serum, CSF, and BAL samples were previously treated with Tris-HCl buffer (0.2 M, pH 8.6) plus dithiothreitol (DTT; 0.003 M) and heated at 56°C for 30 min in order to inactivate rheumatoid factor and dissociate immune complexes. After the reagents were mixed, the slide was gently shaken in an orbital shaker for up to 5 min. Negative-control LA consisted of 25 μl of latex solution added to 25 μl of saline solution. Samples were considered positive when agglutination (clumping) was observed.

### Agglutination score

The LA tests formed loose aggregates within 5 to 10 min in positive assays or remained as a milky suspension in negative assays. Test results were scored as follows: 4+ indicated that the suspension rapidly formed large clumps in a very clear background, mobilizing 100% of SLPs, with formation of a ring; 3+ indicated moderately sized and large clumps were present against a clear background, involving >75% of SLPs, with minor ring formation; 2+ indicated small to moderately sized clumps were present against a slightly cloudy background, mobilizing ≤50% of SLPs, with no ring formation; 1+ indicated fine particle clumping against a cloudy background, usually involving ≤25% of SLPs; and they were scored as negative when there was no visible agglutination. Final readings were made after 10 min. Results were independently scored by two investigators blinded to the identity of the tested serum and the results of other investigators.

### Statistical analysis

Diagnostic values included sensitivity, specificity, positive predictive value (PPV), and negative predictive value (NPV). The receiver operating characteristic (ROC) curves were prepared and analyzed to determine the sensitivity and specificity of each LA assay (gp43-SLPs, mAb17c- SLPs) and DID test. The area under the ROC curve (AUC) was calculated to evaluate the diagnostic value of each LA test. We assumed a test without diagnostic power when the ROC curve was linear with an AUC of 0.5 (i.e., the ROC curve will coincide with the diagonal). On the other hand, a powerful test would provide an AUC of approximately 1.0, indicating the absence of both false-positives and false-negatives (i.e., the ROC curve will reach the upper left corner of the plot). To measure the degree of concordance of the results of the three different assays (gp43-SLPs, mAb17c-SLPs, and DID test), we calculated the kappa statistic and its 95% confidence interval (CI). Kappa values were interpreted as follows: 0.00–0.20, poor agreement; 0.21–0.40, fair agreement; 0.41–0.60, moderate agreement; 0.61–0.80, good agreement; 0.81–1.00, very good agreement [34]. A P-value ≤0.05 was considered as significant. All statistical calculations were performed with MedCalc Statistical Software version 14.8.1 (MedCalc Software bvba, Ostend, Belgium; http://www.medcalc.org; 2014).

## Results

### Detection of anti-gp43 in sera

**Fig 2:**
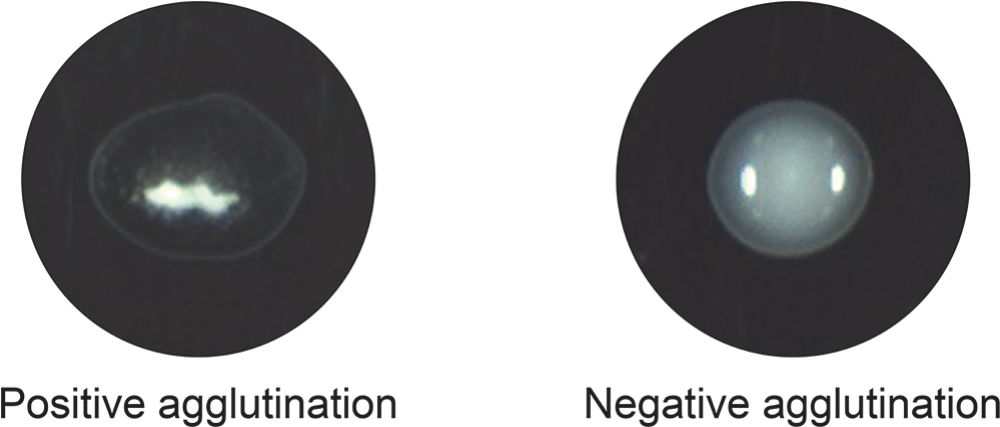
Appearance of typical latex agglutination test. In positive reactions, the LA tests formed loose aggregates within 5 to 10 min (left). In negative reactions, the LA test remained as a milky suspension (right).

**Fig 3:**
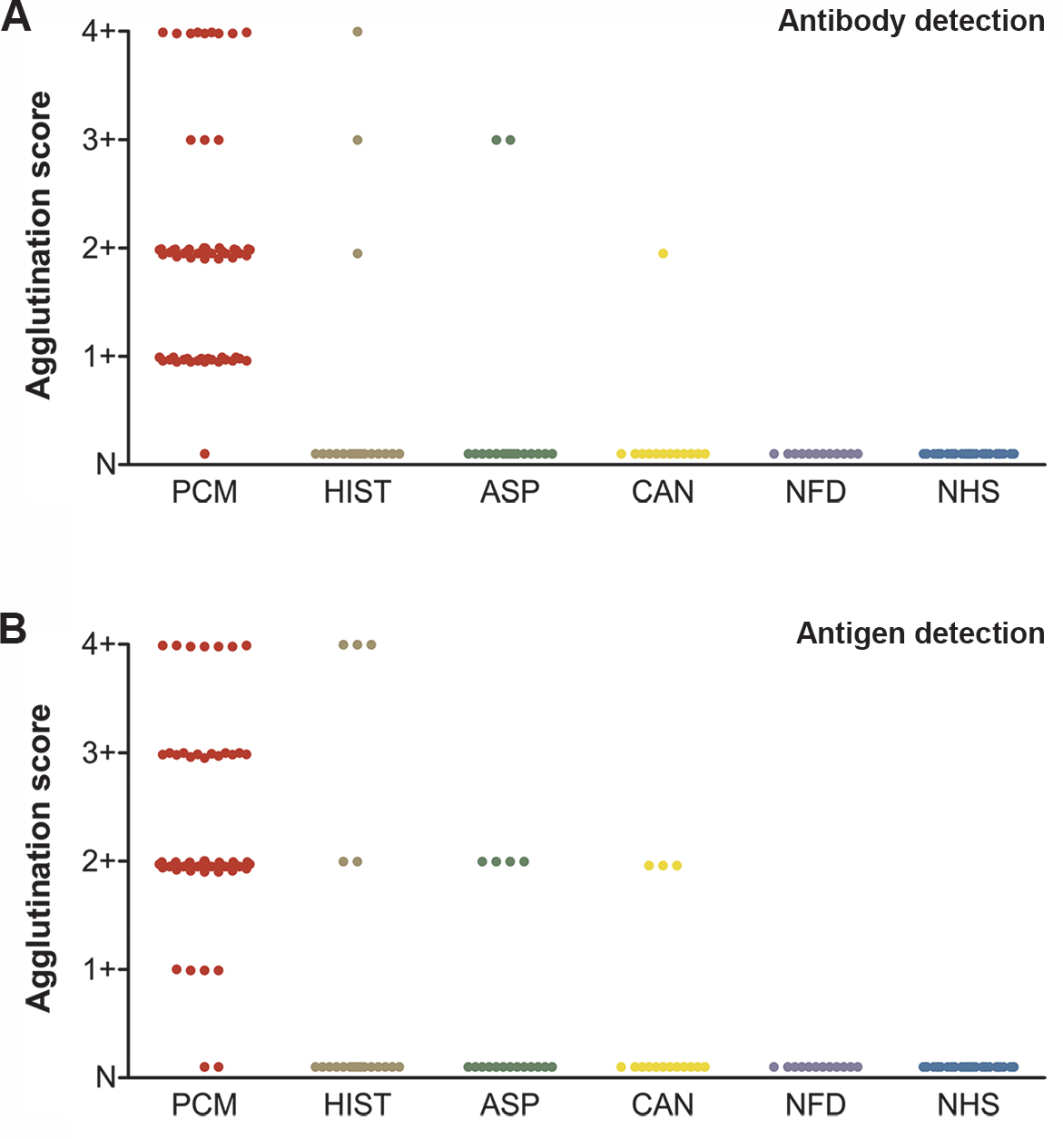
(A) Detection of anti-gp43 antibody in the sera of patients using gp43-SLPs and (B) gp43 antigen in the sera of patients using mAb17c-SLPs. Samples were from paracoccidioidomycosis (PCM; n = 65), histoplasmosis (HIS; n = 18), aspergillosis (ASP; n = 18), candidiasis (CAN; n = 13), no fungal disease (NFD; n = 12), and healthy controls (NHS; n = 38). Agglutination score: (4+) 100%, (3+) 75%, (2+) 50%, (1+) 25%, and (N) negative.

To determine if the proposed test is feasible for detecting specific antibodies against *P*. *brasiliensis*, we performed an LA test with gp43-SLPs using serum specimens from 65 cases of PCM and controls, including 49 cases with fungal infection (histoplasmosis, aspergillosis, candidiasis) and normal healthy subjects (NHS, n = 38; Table 1). Fig. 2 shows an example of positive and negative LA reactions. Among the 65 PCM sera, 64 (98.46%) were positive and 1 (1.53%) was negative. Among the heterologous sera: 3 histoplasmosis sera were positive (16.6%) and 15 (83.3%) negative; 2 aspergillosis sera were positive (11.11%) and 16 (88.8%) negative; 1 candidiasis sera sample was positive (7.7%) and 12 (92.3%) negative. All sera from healthy humans were unreactive (Fig. 3A). The sensitivity, specificity, PPV, and NPV values for gp43-SLPs were 98.46% (95% CI 91.7–100.0), 93.94% (95% CI 87.3–97.7), 91.4%, and 98.9%, respectively.

**Table 1:** Detection of anti-gp43 antibody and gp43 antigen in sera from patients with or without fungal disease.

Serum Samples	Antibody Detection (gp43-SLPs)		Antigen Detection (mAb17c-SLPs)	
	Positive	Negative	Positive	Negative
**PCM**				
Acute (n = 8)	8 (100%)	0	8 (100%)	0
Chronic (n = 57)	56 (98.25%)	1 (1.75%)	55 (96.49%)	2 (3.5%)
Total (n = 65)	64 (98.46%)	1 (1.54%)	63 (96.9%)	2 (3.07%)
**Histoplasmosis** (n = 18)	3 (16.66%)	15 (83.33%)	5 (27.77%)	13 (72.22%)
**Aspergillosis** (n = 18)	2 (11.11%)	16 (88.88%)	4 (22.22%)	14 (77.77%)
**Candidiasis** (n = 13)	1(7.69%)	12(92.3%)	3 (23.07%)	10 (76.92%)
**No fungal diseases** (n = 12)	0	12 (100%)	0	12 (100%)
**NHS** (n = 38)	0	38 (100%)	0	38 (100%)

Data are n(%). PCM, paracoccidioidomycosis; NHS, normal healthy subjects.

### Detection of gp43 antigen in sera

To rate the performance of the LA test for detecting gp43 antigen, the same set of samples (PCM and controls) used for antibody detection were tested using mAb17c-SLPs (Table 1 and Fig. 3B). Among the 65 PCM sera, 63 (96.92%) were positive and 2 (3.08%) were negative. Among the heterologous sera: 5 histoplasmosis sera were positive (27.7%) and 13 (72.2%) were negative; 4 aspergillosis sera were positive (22.2%) and 14 (77.8%) negative; 3 candidiasis sera were positive (23%) and 10 (77%) negative. All sera from healthy people were unreactive, as well as 12 sera samples from patients with no fungal diseases. The sensitivity, specificity, PPV, and NPV values for mAb17c-SLPs were 96.92% (95% CI 89.3–99.6), 88.89% (95% CI 81.0–94.3), 85.1%, and 97.8%, respectively.

### Detection of anti-gp43 in CSF

**Fig 4:**
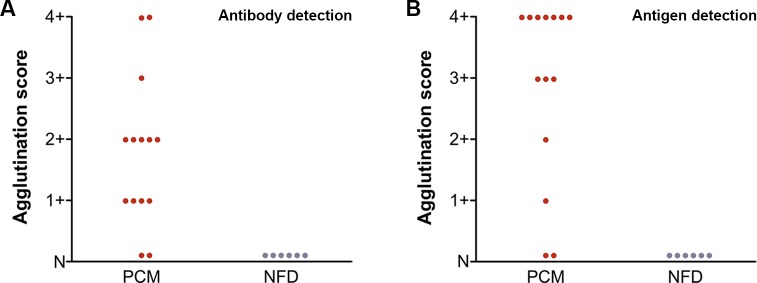
(A) Detection of anti-gp43 antibody in the cerebrospinal fluid of PCM patients using gp43-SLPs and (B) gp43 antigen in the cerebrospinal fluid of PCM patients using mAb17c-SLPs. Agglutination score: (4+) 100%, (3+) 75%, (2+) 50%, (1+) 25%, and (N) negative. Samples were from 14 PCM patients and 6 negative controls with no fungal disease (NFD).

In order to evaluate whether the LA test was feasible for detecting anti-gp43 antibodies in CSF, 14 CSF samples were submitted to the test with gp43-SLPs. Twelve of the 14 CSF samples were positive (Table 2 and Fig. 4A); two samples had agglutination pattern 4+ (100% visible agglutination), 1 had pattern 3+ (75% visible agglutination), 5 had pattern 2+ (50% agglutination visible), 4 had pattern 1+ (25% visible agglutination), and two samples exhibited no visible agglutination (N).

**Table 2:** Detection of anti-gp43 antibody and gp43 antigen in cerebrospinal fluid (CSF) from patients with or without paracoccidioidomycosis (PCM).

CSF Samples	Antibody Detection (gp43-SLPs)		Antigen Detection (mAb17c-SLPs)	
	Positive	Negative	Positive	Negative
**PCM patients** (n = 14)	12 (85.71%)	2 (14.28%)	12 (85.71%)	2 (14.28%)
**No fungal disease patients** (n = 6)	0	6 (100%)	0	6 (100%)

Data are n(%).

### Detection of gp43 in CSF

Previously, we reported that the diagnosis of neuroPCM can be achieved by detection of the gp43 antigen in CSF by ELISA [24]. In this study, when we performed the mAb17c-SLP test to detect the gp43 antigen, we observed that 12 of the 14 samples tested were positive (Table 2 and Fig. 4B). Seven samples had agglutination pattern 4+ (100% visible agglutination), 3 had pattern 3+ (75% of visible agglutination), 1 had pattern 2+ (50% visible agglutination), 1 had pattern 1+ (25% visible agglutination), and two samples exhibited no visible agglutination (N). None of the six samples tested as negative controls were positive.

The LA test for detection of anti-gp43 antibody (gp43-SLPs) or antigen (mAb17c-SLPs) in CSF had a sensitivity of 85.71% (95% CI 57.2–98.2) and specificity of 100% (95% CI 54.1–100.0). The PPV and NPV were 100% and 75%, respectively.

### Detection of anti-gp43 in BAL fluid

**Fig 5:**
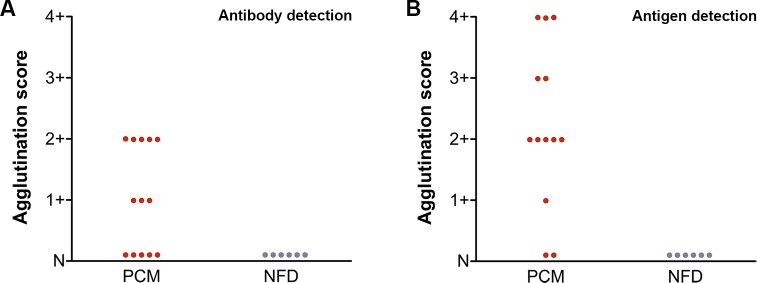
(A) Detection of anti-gp43 antibody in the bronchoalveolar lavage fluid from PCM patients using gp43-SLPs and (B) gp43 antigen in the bronchoalveolar lavage fluid from PCM patients using mAb17c-SLPs. Agglutination score: (4+) 100%, (3+) 75%, (2+) 50%, (1+) 25%, and (N) negative. Samples were from 13 PCM patients and 6 negative controls with no fungal disease (NFD).

A total of 13 BAL fluid samples were investigated for anti-gp43, as it has been successfully used for ELISA [35]. Eight of the tested samples were positive (Table 3 and Fig. 5A). Five samples had agglutination pattern 2+ (50% of visible agglutination), 3 had pattern 1+ (25% of visible agglutination), and 5 exhibited no visible agglutination (N). None of the six samples tested as negative controls were positive. The LA test for detection of anti-gp43 in BAL fluid had a sensitivity of 61.54% (95% CI 31.6–86.1) and specificity of 100% (95% CI 54.1–100.0). The PPV and NPV were 100% and 54.5%, respectively.

**Table 3:** Detection of anti-gp43 antibody and gp43 antigen in bronchoalveolar lavage (BAL) fluid from patients with or without paracoccidioidomycosis (PCM).

BAL Samples	Antibody Detection (gp43-SLPs)		Antigen Detection (mAb17c-SLPs)	
	Positive	Negative	Positive	Negative
**PCM patients** (n = 13)	8 (61.53%)	5 (38.46%)	11 (84.61%)	2 (15.38%)
**No fungal disease patients** (n = 6)	0	6 (100%)	0	6 (100%)

Data are n(%).

### Detection of gp43 in BAL fluid

Eleven of 13 BAL fluid samples were positive for gp43 detection (Table 3 and Fig. 5B), indicating that mAb17c-SLPs have a greater detection capability than gp43-SLPs for this type of biological sample. Three samples had agglutination pattern 4+ (100% visible agglutination), 2 had pattern 3+ (75% agglutination visible), 5 had pattern 2+ (50% visible agglutination), 1 had pattern 1+ (25% visible agglutination), and two samples exhibited no visible agglutination (N). None of the six samples tested as negative controls were positive. The LA test for detection of gp43 antigen in BAL fluid had a sensitivity of 84.62% (95% CI 54.6–98.1) and specificity of 100% (95% CI 54.1–100.0). The PPV and NPV values were 100% and 75%, respectively.

### Serological follow-up for antigen and antibody detection

**Fig 6:**
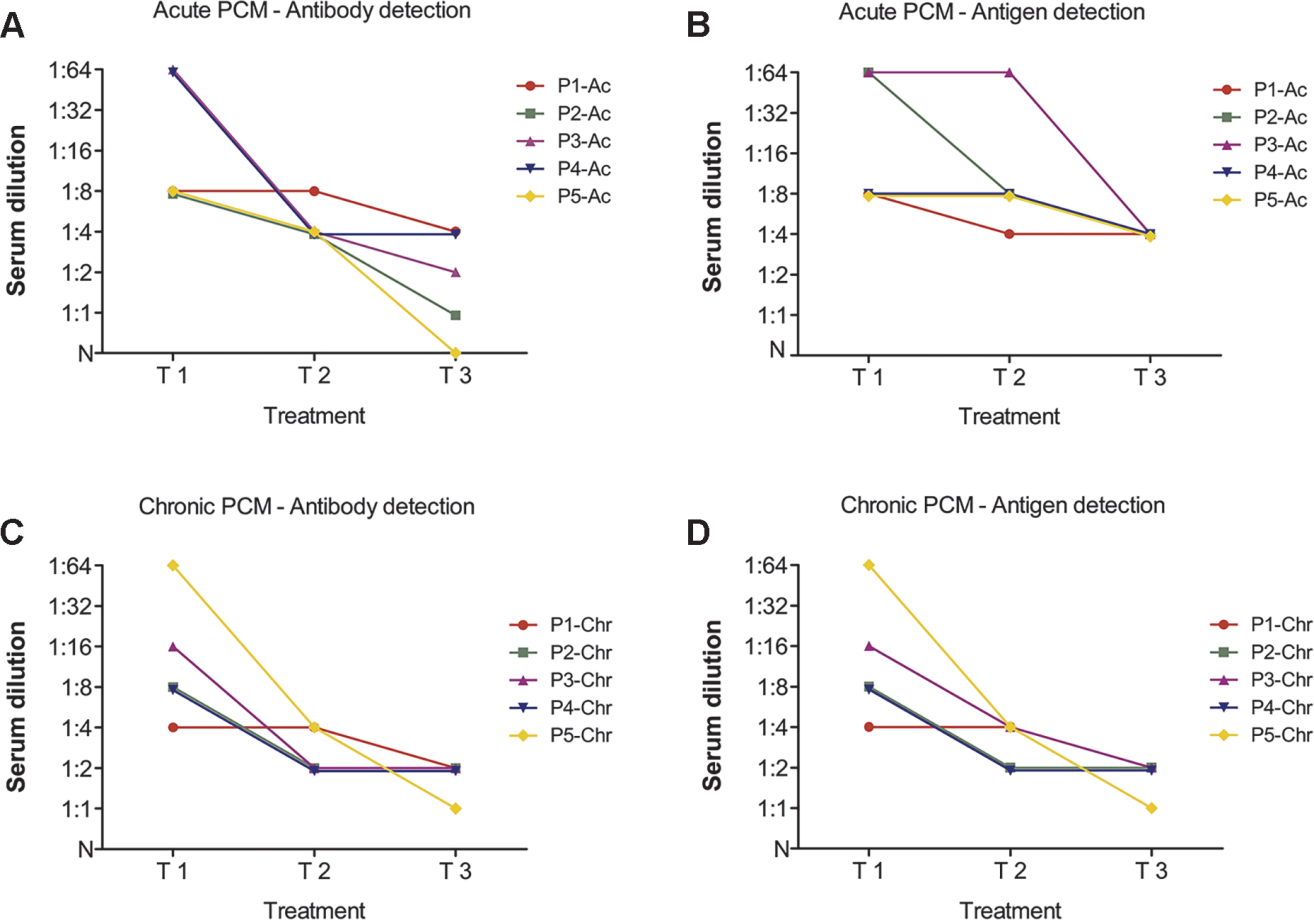
Representative curves for serological follow-up. Samples were from 10 PCM patients with acute (n = 5) or chronic (n = 5) forms of the disease before (T1, at the moment of disease diagnosis), during (T2 = 3 months), and after treatment (T3, between 18–24 months). (**A**, **C)** Anti-gp43 antibody detection using gp43-SLPs and (**B**, **D)** gp43 antigen detection using mAb17c-SLPs.

We were able to assess the efficiency of the LA test in treatment follow-up using sera from patients with confirmed PCM who were undergoing therapy. Fig. 6 shows representative curves for the serological follow-up of 10 PCM patients with acute (n = 5) and chronic (n = 5) forms before (T1), during (T2), and after treatment (T3).

### Statistical analysis using the ROC curve

**Fig 7:**
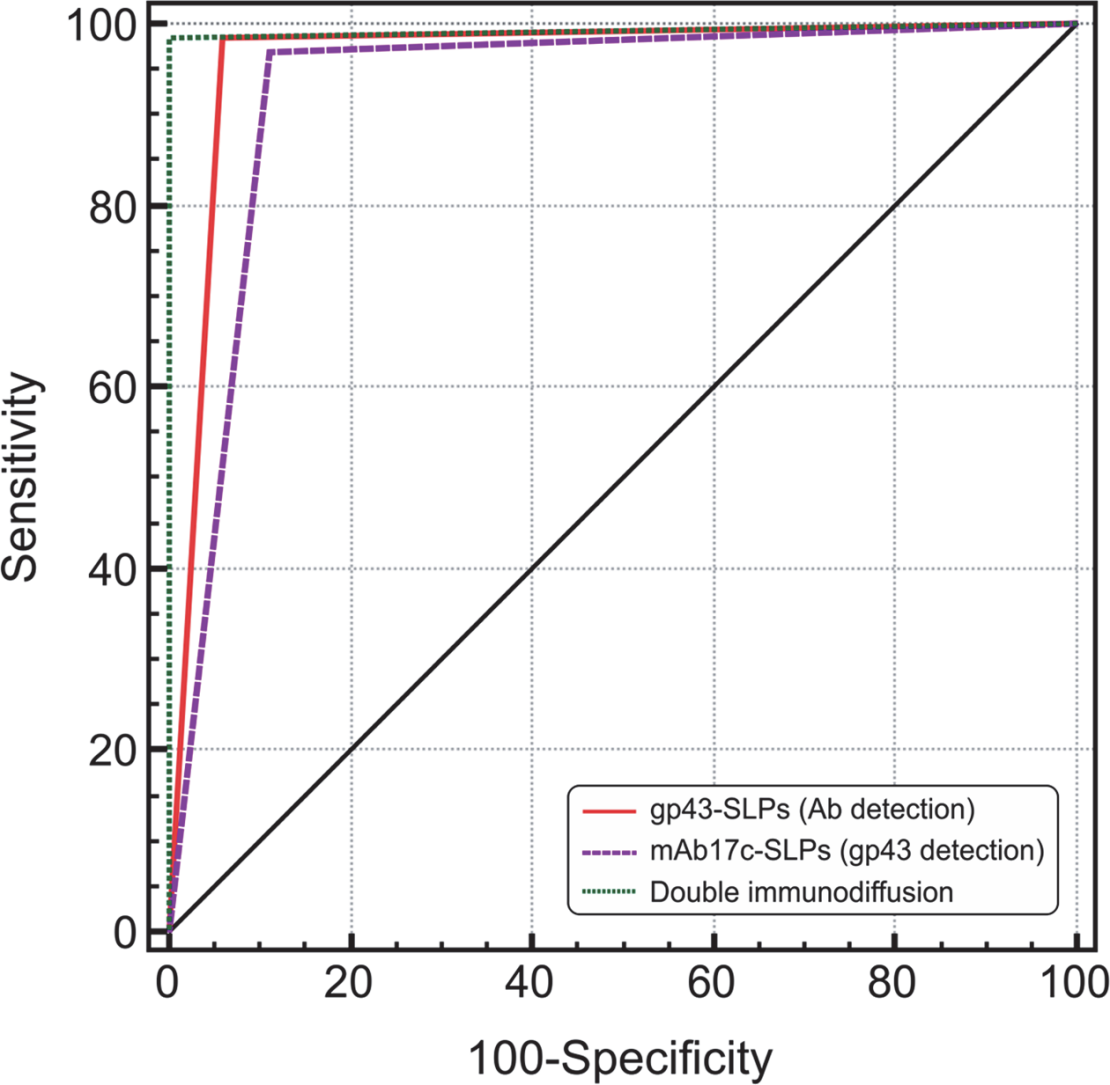
Receiver operating characteristics (ROCs) depicting assay sensitivity and specificity. Samples were from 65 PCM patient sera and 99 control sera, including patients with histoplasmosis, aspergillosis, non-fungal diseases, and healthy subjects. The ROC is plotted between the true-positive rate (sensitivity) on the y-axis and the false-positive rate (100-specificity) on the x-axis. The area under the curve (AUC) represents the accuracy of the LA test, which was 0.962±0.0143 (95% CI 0.920–0.986, P<0.0001) for the gp43-SLPs, 0.929±0.0192 (95% CI 0.878–0.963, P<0.0001) for the mAb17c-SLPs, and 0.992±0.00769 (95% CI 0.964–1.000, P<0.0001) for the DID. The more the AUC is greater than 0.5, the better the test.

Fig. 7 shows a ROC curve depicting assay sensitivity and specificity based on testing 65 PCM patient sera and 99 control sera, including patients with histoplasmosis, aspergillosis, non-fungal diseases, and healthy subjects, indicating optimum performance of the LA test. Our results indicate that the LA test using gp43-SLPs provides a better AUC (0.962±0.0143, 95% CI 0.920–0.986, P<0.0001) than mAb17c-SLPs (0.929±0.0192, 95% CI 0.878–0.963, P<0.0001). As previously reported [11], DID also presented good results (AUC 0.992±0.00769, 95% CI 0.964–1.000, P<0.0001). In addition, we used the Kappa test to assess the agreement between different serological assays (gp43-SLPs, mAb17c-SLPs, and DID), finding very good agreement between gp43-SLPs and DID (*k* = 0.92±0.030, 95% CI 0.865–0.984) and between mAb17c-SLPs and DID (*k* = 0.850±0.041, 95% CI 0.770–0.931), which reinforces the power of our LA test over DID, especially because we were able to diagnosis PCM in less than 10 min but DID required at least 72 hours. The same agreement was observed for the gp43-SLP LA test vs. mAb17c-SLP LA test (*k* = 0.926±0.030, 95% CI 0.868–0.984).

## Discussion

The regions where PCM occurs are primarily vast rural areas where poverty and a poor, sometimes nonexistent, public health system are predominant. Diagnostic centers are scarce and difficult to access for affected individuals. In general, these laboratories have poor infrastructure for serological and mycological diagnosis. Generally, biological samples are collected and sent to research centers that provide specialized diagnosis and treatment options. Therefore, simple serological techniques with the possibility of use in precarious locations should be standardized and implemented in remote rural regions where PCM is endemic.

The LA test has been proposed for the diagnosis of several fungal diseases, including histoplasmosis [36–38], candidiasis [39,40], sporotrichosis [41], aspergillosis [42], and coccidioidomycosis [43], and many other applications are currently emerging [28]. The LA test is a diagnostic method for the detection of antibodies or circulating antigens based on the agglutination of sensitized polystyrene particles. This technical procedure allows rapid diagnosis without prior training or sophisticated equipment.

In this study, the gp43-SLP test for detecting specific anti-gp43 antibodies exhibited high performance and was able to detect antibodies in 98.4% of serum samples. Minor cross-reactions occurred in sera from patients with histoplasmosis (16.6%), aspergillosis (11.11%), and candidiasis (7.7%), but the sensitivity and specificity of the gp43-SLP test indicated that it was successful. Cross-reactivity is common in serological assays that rely on the detection of antibodies among fungal infections due to antigenic similarity [44], especially the shared galactose and mannose epitopes of gp43 in *P*. *brasiliensis* [45]. Using a crude antigen preparation of *P*. *brasiliensis*, Restrepo and Moncada [23] previously showed high cross-reactivity with sera from patients with histoplasmosis, aspergillosis, candidiasis, coccidioidomycosis, and sporotrichosis. Also, more recently, Silveira-Gomes *et al*. [46] reported 84% sensitivity and 81% specificity using SLPs with a pool of exoantigens of *P*. *brasiliensis*. However, cross-reactivity occurred with sera from patients with aspergillosis (27%), histoplasmosis (27%), and non-fungal infections (22%). We demonstrated that the detection of anti-gp43 was very efficient in CSF samples (85.7% positivity), and less efficient in BAL fluid samples (61.5% positivity).

In patients with immune deficiencies who fail to produce normal levels of immunoglobulins, the detection of antibodies is difficult and requires more sensitive techniques. However, very sensitive techniques capable of detecting low levels of antibody may lose specificity. Thus, detection of circulating antigens rather than antibodies may constitute an important tool for the diagnosis or monitoring of patients undergoing treatment, particularly for immunocompromised patients in whom early and accurate diagnosis is mandatory for the implementation of an effective treatment [47].

No studies are yet available on antigen detection using the LA test as a diagnostic method for PCM. In our study, the mAb17c-SLP test performed well and was able to detect antigen in 96.9% of serum samples. Cross-reactions occurred in sera from patients with histoplasmosis (27.7%), aspergillosis (22.2%), and candidiasis (23%). Under these conditions, the sensitivity and specificity indicate that the test was successful for diagnostic purposes. Moreover, detection of gp43 in CSF and BAL fluid samples was efficient. Although, other techniques (e.g., inhibition-ELISA) may detect circulating antigens in PCM sera, they are laborious compared to the LA test and require trained personnel for implementation [24,47].

A basal antibody level may last for years in patients even after the remission of clinical symptoms [48,49], but antigen and antibody titers may be influenced by therapy [50] and the ability to develop an immune response against the pathogen [51]. Patients followed up using the LA test presented with no known comorbidities, and the therapeutic regimen was based on daily itraconazole or classical treatment with sulfamethoxazole plus trimethoprim. Judging from the positive agglutination using gp43-SLPs or mAb17c-SLPs, distinct titers were observed at the diagnosis of patients with the chronic and acute forms (Fig. 6, T1). We observed a decreasing antigen titer during the first period of treatment (T2), but at the end of the treatment (T3) none of the patients with acute (Fig. 6B) or chronic forms (Fig. 6D) had a negative test, and they returned to basal antigens titers. From the perspective of antibody titration using gp43-SLPs, most sera had a considerable decrease in the titer of anti-gp43 antibodies. At the end of the treatment, the antibody titers were negative to 1:4 (Fig. 6A and C), which is in agreement with other serological tests [24–26].

With the recent introduction of dissimilar species as etiological agents of PCM [5,9] with different antigenic composition [11,52,53], the serological tests may develop towards taxonomic advances and offer alternative antigenic preparations for rapid and accurate patient diagnosis. For a long time, gp43 has been highlighted as the immunodominant antigen of PCM due to *P*. *brasiliensis sensu lato* [27]. However, experimental evidence from our group shows that patients infected with *P*. *lutzii* may not react with *P*. *brasiliensis* gp43 [10,12,52,53], probably due to the lack of identity in antigenic epitopes or the absence or minimal expression of *P*. *lutzii* gp43 during host-parasite interplay [12]. Recently, we proposed an antigenic preparation derived from cell-free *P*. *lutzii* antigens (CFA-Pl) that successfully diagnosed PCM with high sensitivity and specificity due to the offshoot *P*. *lutzii* [11]. The utility of this CFA-Pl preparation for LA tests is currently being investigated in our laboratory and may help diagnose possible false-negatives using gp43 as the main antigenic component.

In conclusion, due to the high diagnostic accuracy, low cost in terms of production of the antigen, reduced assay time, simplicity, and availability, this LA test may have great potential for the diagnosis of PCM due to *P*. *brasiliensis* complex (S1, PS2, and PS3) in endemic regions of Latin America. The implementation of simple diagnostic tests in remote areas is preferred to tests that use molecular biology techniques that, although effective, are extremely difficult or even impossible to implement. The LA test is an especially useful screening method and valid in both field research, where there is adequate space and equipment available for diagnosis, and hospital screening. Due to its advantages, the test should be used in clinical laboratories as a new diagnostic method.

## Supporting Information

S1 Checklist STARD Checklist. (DOC)

S1 Flow Diagram STARD Flow Diagram. (PDF)
