# Supplementary figures and images for: Immunodiagnosis of Paracoccidioidomycosis due to Paracoccidioides brasiliensis Using a Latex Test: Detection of Specific Antibody Anti-gp43 and Specific Antigen gp43
Source: PLoS Negl Trop Dis. 2015 Feb 13;9(2):e0003516. doi: 10.1371/journal.pntd.0003516 (PMC4334539; doi:10.1371/journal.pntd.0003516)

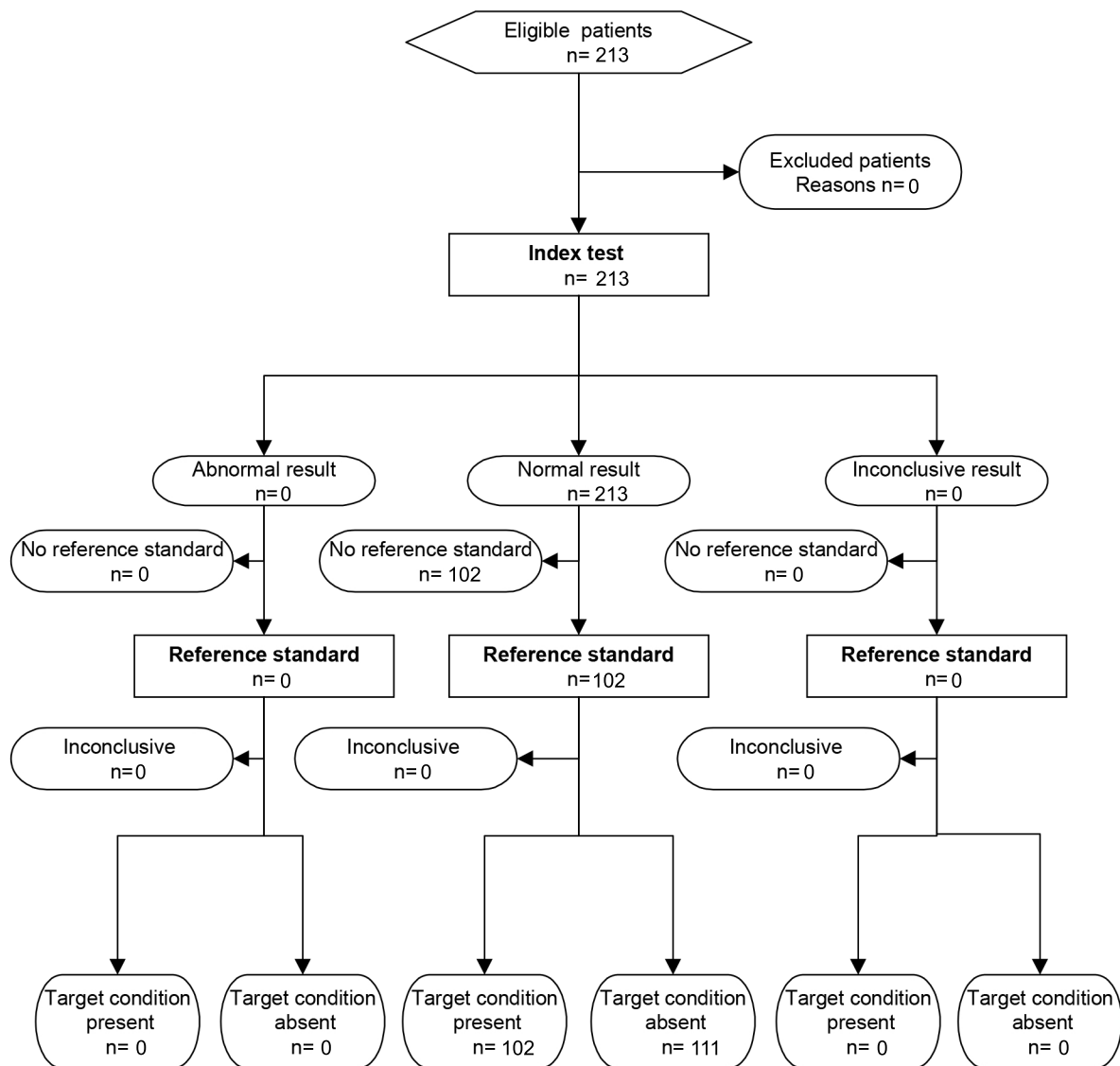

Supplement: S1 Flow Diagram — (PDF) [file pntd.0003516.s002.pdf]
